# Emergency Department Triage, Transfer Times, and Hospital Mortality of Patients Admitted to the ICU: A Retrospective Replication and Continuation Study*

**DOI:** 10.1097/CCM.0000000000006396

**Published:** 2024-08-19

**Authors:** Michael C. van Herwerden, Carline N. L. Groenland, Fabian Termorshuizen, Wim J. R. Rietdijk, Fredrike Blokzijl, Berry I. Cleffken, Tom Dormans, Jelle L. Epker, Lida Feyz, Niels Gritters van den Oever, Pim van der Heiden, Evert de Jonge, Gideon H. P. Latten, Ralph V. Pruijsten, Özcan Sir, Peter E. Spronk, Wytze J. Vermeijden, Peter van Vliet, Nicolette F. de Keizer, Corstiaan A. den Uil

**Affiliations:** 1 Department of Intensive Care Medicine, Erasmus MC, University Medical Center, Rotterdam, The Netherlands.; 2 Department of Medical Informatics, Amsterdam University Medical Center, Amsterdam, The Netherlands.; 3 National Intensive Care Evaluation (NICE) Foundation, Amsterdam, The Netherlands.; 4 Vrije Universiteit, Amsterdam, The Netherlands.; 5 Department of Critical Care, University Medical Center Groningen, Groningen, The Netherlands.; 6 Department of Intensive Care Medicine, Maasstad Hospital, Rotterdam, The Netherlands.; 7 Department of Intensive Care Medicine and Emergency Department, Zuyderland, Sittard-Geleen, The Netherlands.; 8 Department of Cardiology, Erasmus MC, University Medical Center, Rotterdam, The Netherlands.; 9 Department of Intensive Care Medicine, Treant, Emmen, The Netherlands.; 10 Department of Intensive Care Medicine, Reinier de Graaf Gasthuis, Delft, The Netherlands.; 11 Department of Intensive Care Medicine, University Medical Center Leiden, Leiden, The Netherlands.; 12 Department of Intensive Care Medicine, Ikazia Hospital, Rotterdam, The Netherlands.; 13 Department of Emergency Medicine, Radboud University Medical Center, Nijmegen, The Netherlands.; 14 Department of Intensive Care Medicine, Gelre Hospital, Apeldoorn, The Netherlands.; 15 Department of Intensive Care Medicine, Medical Spectrum Twente, Twente, The Netherlands.; 16 Department of Intensive Care Medicine, Haaglanden Medical Center, Den Haag, The Netherlands.

**Keywords:** critically ill, emergency department, intensive care unit, length of stay, mortality

## Abstract

**OBJECTIVES:**

This study aimed to provide new insights into the impact of emergency department (ED) to ICU time on hospital mortality, stratifying patients by academic and nonacademic teaching (NACT) hospitals, and considering Acute Physiology and Chronic Health Evaluation (APACHE)-IV probability and ED-triage scores.

**DESIGN, SETTING, AND PATIENTS:**

We conducted a retrospective cohort study (2009–2020) using data from the Dutch National Intensive Care Evaluation registry. Patients directly admitted from the ED to the ICU were included from four academic and eight NACT hospitals. Odds ratios (ORs) for mortality associated with ED-to-ICU time were estimated using multivariable regression, both crude and after adjusting for and stratifying by APACHE-IV probability and ED-triage scores.

**INTERVENTIONS:**

None.

**MEASUREMENTS AND MAIN RESULTS:**

A total of 28,455 patients were included. The median ED-to-ICU time was 1.9 hours (interquartile range, 1.2–3.1 hr). No overall association was observed between ED-to-ICU time and hospital mortality after adjusting for APACHE-IV probability (*p* = 0.36). For patients with an APACHE-IV probability greater than 55.4% (highest quintile) and an ED-to-ICU time greater than 3.4 hours the adjusted OR (ORs_adjApache_) was 1.24 (95% CI, 1.00–1.54; *p* < 0.05) as compared with the reference category (< 1.1 hr). In the academic hospitals, the ORs_adjApache_ for ED-to-ICU times of 1.6–2.3, 2.3–3.4, and greater than 3.4 hours were 1.21 (1.01–1.46), 1.21 (1.00–1.46), and 1.34 (1.10–1.64), respectively. In NACT hospitals, no association was observed (*p* = 0.07). Subsequently, ORs were adjusted for ED-triage score (ORs_adjED_). In the academic hospitals the ORs_adjED_ for ED-to-ICU times greater than 3.4 hours was 0.98 (0.81–1.19), no overall association was observed (*p* = 0.08). In NACT hospitals, all time-ascending quintiles had ORs_adjED_ values of less than 1.0 (*p* < 0.01).

**CONCLUSIONS:**

In patients with the highest APACHE-IV probability at academic hospitals, a prolonged ED-to-ICU time was associated with increased hospital mortality. We found no significant or consistent unfavorable association in lower APACHE-IV probability groups and NACT hospitals. The association between longer ED-to-ICU time and higher mortality was not found after adjustment and stratification for ED-triage score.

**KEYPOINTS**

**Question**: What is the impact of “emergency department (ED)-to-ICU time” on hospital mortality while considering the influence of disease severity (using Acute Physiology and Chronic Health Evaluation [APACHE]-IV probability and ED-triage scores) in subgroups of patients treated in academic and nonacademic teaching hospitals?

**Findings**: Prolonged ED-to-ICU time (> 1.6 hr) was associated with increased hospital mortality among patients in academic hospitals with the highest APACHE-IV probability scores. Interestingly, no association was observed in nonacademic teaching hospitals and patients with lower APACHE-IV probability scores.

**Meaning**: Healthcare personnel in academic hospitals should be aware of the negative impact of prolonged ED-to-ICU time.

Increased patient volume and limited ICU capacity has resulted in prolonged emergency department (ED) length of stay (LOS) (1–6). ED-to-ICU time and ED-boarding time describe throughput times (4, 7). ED-to-ICU time is the duration from ED admission to ICU admission. ED-boarding time is the period between the decision to admit a patient to the ICU and their actual transfer, during which they remain in the ED.

Prior research indicates that prolonged ED-to-ICU time (8) or ED-boarding time (9) may contribute to higher ICU and hospital mortality rates. Some EDs have addressed this by incorporating ICU beds, which has led to reduced mortality (10).

In an Australasian study (11), no significant association was found between ED-to-ICU time and hospital mortality. However, the analysis had limitations: a prolonged median ED-to-ICU time of 3.9 hours, limited consideration of confounders, and no stratification by disease severity. Given these conflicting findings, the association between ED LOS and mortality remains a topic of discussion.

In 2019, our research group conducted a retrospective cohort study in academic hospitals. The study found that prolonged ED-to-ICU times were associated with increased mortality, particularly among patients with the highest Acute Physiology and Chronic Health Evaluation (APACHE)-IV probability (8).

A critique of this study concerned the role of the APACHE-IV score that may act as a mediator instead of a confounder. This raised questions about the appropriateness of adjusting for the APACHE-IV probability, an estimated mortality risk based on the APACHE-IV score (12, 13). The APACHE-IV score was assessed upon ICU admission, after time spent in the ED. It was argued that adjusting for APACHE-IV probability might obscure a potentially positive effect of a prolonged ED stay, especially if intensive ED treatment stabilizes physiologic parameters used in the APACHE-IV model (14). Conversely, adjusting for the APACHE-IV probability might weaken the unfavorable effect of a prolonged ED stay, if this stay implies an undesirable delay to more appropriate ICU treatment. However, a significant reversal occurred from a favorable crude association to an unfavorable one between ED-to-ICU time and mortality after adjusting for the APACHE-IV probability. This was more pronounced among patients with a higher APACHE-IV probability, validating the APACHE-IV score as an early indicator of clinical condition. This challenges the idea that the APACHE-IV score acts solely as a mediator. Nevertheless, we agree that the APACHE-IV score may reflect the effects of time and treatment at the ED. It may be useful to include a measure of disease severity earlier in the treatment trajectory (15).

Given our prior academic hospital focused study (8) and concerns about the suitability of APACHE-IV probability, we performed a retrospective replication and continuation study with the primary objective of reexamining the associations between ED-to-ICU time and hospital mortality after ICU admission in a larger cohort. We included subgroup analyses categorizing patients treated in academic and in nonacademic teaching (NACT) hospitals. In addition to the APACHE-IV probability, an adjustment and stratification based on the ED-triage score was performed. Our hypothesis is that the ED-triage score at ED admission may provide an earlier and potentially more relevant measure of illness severity upon ICU admission compared with the APACHE-IV probability.

## MATERIALS AND METHODS

### ED and ICU Registry

A retrospective cohort study was conducted using data from the Dutch quality registry National Intensive Care Evaluation (NICE). The NICE registry includes information from all admissions to Dutch ICUs (*n* = 79) (16). It contains demographics, physiologic parameters, comorbidities, diagnostics including APACHE-IV probability, and patients’ pre-ICU locations (e.g., ED, operation room, and ward). Additionally, it records ICU admission reasons and mortality rates. Temporal data for ICU admission decisions are not registered.

All academic (*n* = 7) and NACT hospitals with a minimum of 12 ICU beds (*n* = 28) were invited to participate. ED admission date, time (physical arrival), and triage scores, which are not available in the NICE registry, were retrospectively collected from medical records. The ED-to-ICU time was defined as the duration between the patient’s physical arrival at the ED and their admission to the ICU, divided into quintiles for analysis.

The APACHE-IV probability, encompassing the APACHE-III score, age, comorbidities, acute physiology score within 24 hours of ICU admission, ICU diagnosis, and ICU admission category, estimates the likelihood of mortality in the hospital following ICU treatment (17). For analysis, we divided the APACHE-IV probability into quintiles, merging the first and second quintiles due to infrequent events. This resulted in four groups: less than 8.0%, 8.0–20.4%, 20.4–55.4%, and greater than 55.4%.

The Manchester Triage System (MTS) and Dutch Triage Standard (DTS) are used for initial patient assessments and determining the corresponding treatment resource requirement in the ED (18, 19). These systems assign triage scores based on presenting complaints, anamnestic data, and vital signs to determine the urgency of physician care. To improve comparability, we reclassified the DTS into five severity levels, matching the MTS structure. According to convention, patients with triage scores need physician assessment at specific intervals: red (immediate), orange (10 min), yellow (60 min), and green/blue (120 min). Patients with green and blue triage scores were merged due to anticipated low ICU admissions.

### Dutch Emergency Healthcare Landscape

EDs are found in academic, NACT, and community hospitals nationwide, staffed mainly by emergency physicians who complete a 3-year training. EDs have a mean LOS of 146 minutes (sd +49 min) and handle between 15,000 and 30,000 patients annually (20, 21). While vital for diagnosis and stabilization, EDs lack resources for prolonged organ-support and extended admissions.

Patients needing organ-support care are promptly transferred to the ICU, resulting in short ED-to-ICU times. Here, intensivists oversee treatment and additional interventions like renal replacement therapy and extracorporeal life support (ECLS) can be managed. ICU staff is trained to interpret critical parameters, with a patient-nurse ratio of 1:1 to 2:1 (22).

ICUs are organized within networks per Dutch regulations, facilitating peer review among larger ICUs (≥ 12 beds, in academic and NACT hospitals) and smaller ICUs (< 12 beds, in community hospitals). Academic hospitals specialize in advanced care for specific patient cohorts (e.g., transplant recipients and severe trauma patients). NACT hospitals offer standard care for common medical conditions, some providing additional services like percutaneous coronary interventions (PCIs) and burn patient treatment (22). Both hospital types employ junior doctors and physicians in training, with specialist training primarily completed in academic hospitals.

### Study Population

All patients directly transferred from the ED to the ICU between January 1, 2009, and January 1, 2020, were eligible. Four academic and eight NACT hospitals provided ED admission time, date, and triage scores. **eTable 1** (http://links.lww.com/CCM/H570) displays general information on patient categories and treatments in the participating ICUs. The Erasmus Medical Center Institutional Review Board (EMC-IRB) approved this study (EMC-IRB No. MEC 2020-0272, “Emergency department to ICU time and triage classification as factors for explaining mortality,” approved May 14, 2020) and waived the need for informed consent. Study procedures were in accordance with the ethical standards of the EMC-IRB and with the Helsinki Declaration of 1975.

### Primary and Secondary Outcomes

The primary outcome assessed was hospital mortality, with ICU mortality being the secondary outcome.

### Statistical Analysis

Data were analyzed in six steps. First, the sample was described using medians (interquartile ranges [IQRs]) for continuous variables or number (%) for categorical variables. Second, statistical tests were used to compare the baseline characteristics across the five quintiles for ED-to-ICU times. The Kolmogorov-Smirnov test was used to analyze normality. Continuous variables were compared using analysis of variance for normally distributed data or the Kruskal-Wallis test for non-normally distributed data. Categorical variables were compared using the chi-square test or Fisher exact test.

Third, a binary logistic regression model was used for the analysis of hospital mortality. The ED-to-ICU time was divided into five quintiles, with the quintile of the shortest ED-to-ICU time serving as the reference. All models were adjusted for the particular hospital by including a dummy variable. Odds ratios (ORs) and 95% CIs for the association between ED-to-ICU time quintiles and hospital and ICU mortality were calculated. Fourth, these ORs were adjusted for disease severity using the APACHE-IV probability (ORs_adjApache_) and, in a separate model, using ED-triage score (ORs_adjED_). A Wald test was used to assess statistical significance of the differences in hospital mortality across the five ED-to-ICU time quintiles.

Fifth, we evaluated whether the effect of ED-to-ICU time on hospital mortality was modified by the APACHE-IV probability or ED-triage score. To account for potential interaction among these variables, terms for interaction (APACHE-IV probability or ED-triage score × ED-to-ICU time) were included in the model. This assessment aimed to explore whether APACHE-IV probability and/or ED-triage score modifies the relationship between ED-to-ICU time and hospital mortality. The Wald test was used to assess differences in hospital mortality across the five ED-to-ICU time quintiles, but now within each category of APACHE-IV probability and ED-triage score separately.

Finally, the cohort was divided into academic and NACT hospitals for subgroup analyses. The same model structure was used for the analysis for ICU mortality.

All statistical analyses were conducted using R-Studio (Version 4.2.0; Foundation for Statistical Computing, Vienna, Austria). The *p* values of less than 0.05 were considered statistically significant.

## RESULTS

### Population and Baseline Characteristics

During the period from January 1, 2009, to January 1, 2020, a total of 32,072 patients were transferred directly from the ED to the ICU. Excluding patients with invalid or nonretrievable ED-to-ICU times (*n* = 3,617) resulted in 28,455 patients (88.7%) for the final analysis. **Table 1** presents baseline and in-hospital characteristics. Of these patients, 22,068 (77.6%) had a registered ED-triage score. Baseline data for patients with missing ED-triage scores are in **eTable 2** (http://links.lww.com/CCM/H570).

**TABLE 1.** Baseline and In-Hospital Characteristics of the Total Cohort

Baseline Characteristics	All Patients, *n* = 28,455	ED to ICU Time < 1.1 hr, *n* = 5,826	ED to ICU Time 1.1–1.6 hr, *n* = 5,440	ED to ICU Time 1.6–2.3 hr, *n* = 5,811	ED to ICU Time 2.3–3.4 hr, *n* = 5,711	ED to ICU Time > 3.4 hr, *n* = 5,667	*p*
Age, yr, median (IQR)	61 (47–72)	61 (46–72)	60 (45–72)	60 (45–72)	61 (47–72)	63 (49–74)	< 0.001
Male, gender, *n* (%)	16,752 (58.9)	3,413 (58.6)	3,254 (59.8)	3,512 (60.4)	3,341 (58.5)	3,232 (57.0)	0.006
APACHE-IV score, median (IQR)	60 (38–89)	70 (45–100)	67 (42–97)	62 [39–93)	55 (36–82)	51 (34–73)	< 0.001
APACHE-IV predicted mortality, median (IQR)	0.13 (0.04–0.43)	0.19 (0.05–0.61)	0.18 (0.04–0.56)	0.15 (0.04–0.51)	0.10 (0.03–0.32)	0.08 (0.03–0.22)	< 0.001
ED-triage score available, *n* (%)	22,068 (77.6)	4,194 (72.0)	4,156 (76.4)	4,447 (76.5)	4,534 (79.4)	4,737 (83.5)	
ED-triage score missing, *n* (%)	6,387 (22.4)	1,632 (28.0)	1,284 (23.6)	1,364 (23.5)	1,177 (20.6)	930 (16.5)	
ED-triage score^a^							
Red	8,623 (39.1)	2,407 (57.4)	2,186 (52.6)	1,957 (44.0)	1,322 (29.2)	751 (15.9)	< 0.001
Orange	10,013 (45.4)	1,465 (34.9)	1,639 (39.4)	1,962 (44.1)	2,352 (51.9)	2,595 (54.8)	< 0.001
Yellow	2,938 (13.3)	252 (6.0)	258 (6.2)	461 (10.4)	763 (16.8)	1,204 (21.2)	< 0.001
Green and blue	494 (2.2)	70 (1.7)	73 (1.8)	67 (1.5)	97 (2.1)	187 (3.9)	< 0.001
Hospital type							
Academic	10,279 (36.1)	1,307 (22.4)	2,057 (37.8)	2,325 (40.0)	2,121 (37.1)	2,469 (43.6)	< 0.001
Nonacademic	18,176 (63.9)	4,519 (77.6)	3,383 (62.2)	3,486 (60)	3,590 (62.9)	3,198 (56.4)	< 0.001
Most common admission diagnoses, *n* (%)^b^							
Cardiac arrest	3,818 (13.4)	1,198 (20.6)	969 (17.8)	961 (16.5)	546 (9.6)	144 (2.5)	< 0.01
Trauma (nonoperative)	3,505 (12.3)	497 (8.5)	736 (13.5)	715 (12.3)	753 (13.2)	804 (14.2)	< 0.01
Intracranial/subdural/epidural hemorrhage	1,630 (5.7)	433 (7.4)	363 (6.7)	370 (6.4)	255 (4.5)	209 (3.7)	< 0.01
Respiratory failure	2,687 (9.4)	614 (10.5)	483 (8.9)	504 (8.7)	525 (9.2)	561 (9.9)	< 0.01
Overdose	3,128 (11.0)	755 (13.0)	674 (12.4)	708 (12.2)	621 (10.9)	370 (6.5)	< 0.01
Sepsis	1,927 (6.8)	230 (3.9)	258 (4.7)	359 (6.2)	510 (8.9)	570 (10.1)	< 0.01
Pneumonia	2,379 (8.4)	498 (8.5)	439 (8.1)	456 (7.8)	525 (9.2)	461 (8.1)	0.08
Trauma (operative)	382 (1.3)	40 (0.7)	59 (1.1)	73 (1.3)	100 (1.8)	110 (1.9)	< 0.01
Acute coronary syndrome	384 (1.3)	71 (1.2)	60 (1.1)	85 (1.5)	74 (1.3)	94 (1.7)	0.09
Aneurysm	325 (1.1)	58 (1.0)	51 (0.9)	59 (1.0)	55 (1.0)	102 (1.8)	< 0.01
In-hospital characteristics							
ED to ICU time, hr, median (IQR)	1.9 (1.2–3.1)	0.8 (0.6–0.9)	1.3 (1.2–1.5)	1.9 (1.7–2.1)	2.8 (2.5–3.1)	4.6 (3.9–5.8)	< 0.001
ICU LOS, d, median (IQR)	1.5 (0.7–3.6)	1.6 (0.7–3.9)	1.6 (0.7–4.0)	1.6 (0.7–3.8)	1.4 (0.7–3.2)	1.4 (0.7–2.8)	< 0.001
Hospital LOS, d, median (IQR)	6.8 (2.2–14.0)	6.0 (2.0–14.0)	6.0 (2.0–13.6)	6.1 (2.0–13.0)	7.0 (3.0–14.0)	7.2 (3.1–14.0)	< 0.001
ICU mortality, *n* (%)	4,427 (15.6)	1,196 (20.5)	1,017 (18.7)	1,002 (17.2)	721 (12.6)	491 (8.7)	< 0.001
Hospital mortality, *n* (%)	5,539 (19.5)	1,437 (24.7)	1,218 (22.4)	1,233 (21.2)	923 (16.2)	727 (12.8)	< 0.001

APACHE = Acute Physiology and Chronic Health Evaluation, ED = emergency department, IQR = interquartile range, LOS = length of stay.

a Percentages were calculated based on admissions where an ED-triage score was available (*n* = 22,068).

b Only the ten most reported diagnose groups are reported.

The median age was 61 years (IQR, 47–72 yr) with 58.9% being male. Sixty-three percent of the patients were treated in a NACT hospital (*n* = 18,176). The median APACHE-III score was 60 (IQR, 38–89), and was higher in academic hospitals. Thirty-nine percent of the cases were triaged red. APACHE-IV probability and ED-triage score showed an association (*p* < 0.001) (**eTable 3**, http://links.lww.com/CCM/H570). Most frequent admission diagnoses were cardiac arrest (13.4%), trauma (nonoperative) (12.3%), and overdose (11.0%). Median ED-to-ICU time was 1.9 hours (IQR, 1.2–3.1 hr) and was longer in academic hospitals. Median LOS in the ICU and the hospital were 1.5 days (IQR, 0.7–3.6 d) and 6.8 days (IQR, 2.2–14.0 d), respectively. The overall ICU and hospital mortality rates were 15.6% and 19.5%, respectively. See **eTables 4** and **5** (http://links.lww.com/CCM/H570) for baseline and in-hospital characteristics of the academic and NACT cohorts.

### Primary Outcome: Hospital Mortality Adjusted for APACHE-IV Probability

#### Total Cohort

Without adjustment for disease severity a negative association between longer ED-to-ICU times and hospital mortality was found (*p* < 0.01), with all ORs less than 1.0. The ORs and their corresponding 95% CIs are summarized in **eTable 6**, **Model A** (http://links.lww.com/CCM/H570).

After adjusting for disease severity using the APACHE-IV probability, the consistent trend of lower ORs with longer ED-to-ICU times shifted to an opposite trend of higher values with longer ED-to-ICU times (**eTable 6**, **Model B**, http://links.lww.com/CCM/H570). For the quintile with ED-to-ICU times greater than 3.4 hours, the ORs_adjApache_ was 1.06 (95% CI, 0.94–1.19).

Subsequently, we tested whether APACHE-IV probability, included as an effect modifier, influenced the relationship between ED-to-ICU time and hospital mortality (**eTable 6**, **Models C–F**, http://links.lww.com/CCM/H570). Patients with the highest APACHE-IV probabilities (> 55.4%) and ED-to-ICU times greater than 3.4 hours had an ORs_adjApache_ of 1.24 (95% CI, 1.00–1.54).

#### Academic Hospitals

**Figure 1.**
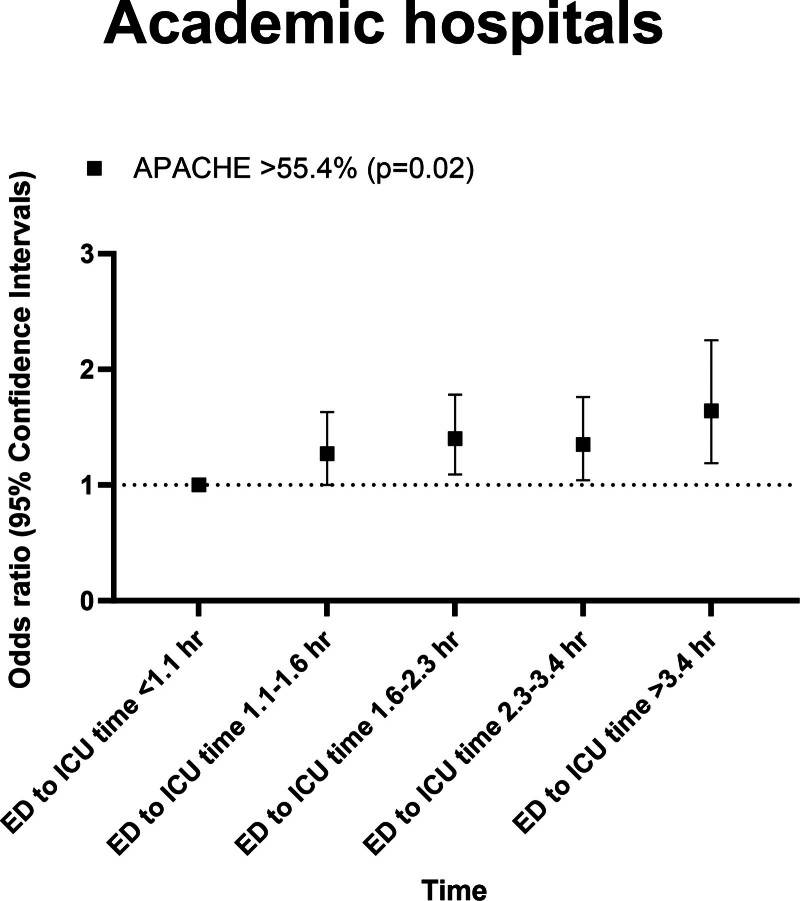
Odds ratios (ORs) for hospital mortality per length of stay in the emergency department (ED), plotted for the Acute Physiology and Chronic Health Evaluation (APACHE) IV probability quintile greater than 55.4% in the academic cohort. The *p* value represents whether ED-to-ICU time as a whole is associated with hospital mortality. For the individual OR and 95% CIs, we refer the reader to eTable 7 (http://links.lww.com/CCM/H570).

Within the academic hospital cohort, higher ORs_adjApache_ were observed with longer ED-to-ICU times. The ORs_adjApache_ for an ED-to-ICU time of greater than 3.4 hours was 1.34 (95% CI, 1.10–1.64). Detailed results can be found in **eTable 7** and **eFigure 1**, **upper row** (http://links.lww.com/CCM/H570). Patients with the highest APACHE-IV probabilities (> 55.4%) demonstrated an ORs_adjApache_ of 1.64 (95% CI, 1.19–2.25) for the greater than 3.4 hours time interval (**Fig. 1**).

#### Nonacademic Teaching Hospitals

In the NACT hospitals, the ORs_adjApache_ for hospital mortality within different time intervals ranged between 0.83 (95% CI, 0.72–0.95) for the 1.1–1.6 hours quintile and 0.92 (95% CI, 0.79–1.08) for the greater than 3.4 hours quintile (eTable 7, http://links.lww.com/CCM/H570). When exploring the interaction between APACHE-IV probability and time intervals across all APACHE-IV probability groups, none of the ORs_adjApache_ reached a lower 95% CI boundary greater than 1.0 (**eFig. 1**, **lower row**, http://links.lww.com/CCM/H570).

### Primary Outcome: Hospital Mortality Adjusted for ED Triage

#### Total Cohort

Following the adjustment for the ED-triage score, all ORs_adjED_ within quintiles of increasing ED-to-ICU times were less than 1.0. These ORs and their corresponding 95% CIs are summarized in **eTable 8**, **Model B** (http://links.lww.com/CCM/H570).

Patients assigned to the blue/green and yellow triage groups, with an ED-to-ICU time of 2.3–3.4 hours, as well as patients in the blue/green, yellow, and orange triage groups with an ED-to-ICU time greater than 3.4 hours, exhibited ORs_adjED_ below 1, with their 95% CIs also less than 1. Conversely, within the red-triaged patient group, those with an ED-to-ICU time of 1.6–2.3 hours demonstrated an ORs_adjED_ of 1.15 (95% CI, 0.99–1.31) (**eTable 8**, **Models C–F**, http://links.lww.com/CCM/H570).

#### Academic Hospitals

Patients with a red-triage score and an ED-to-ICU time of 1.6–2.3 and 2.3–3.4 hours, had an ORs_adjED_ for hospital mortality of 1.31 (95% CI, 1.07–1.62) and 1.37 (95% CI, 1.08–1.73), respectively (**eTable 9** and **eFig. 2**, **upper row**, http://links.lww.com/CCM/H570).

#### Nonacademic Teaching Hospitals

In the NACT hospitals, all sequential time intervals exhibited ORs_adjED_ less than 1, with their 95% CIs also less than 1 (**eFig. 2**, **lower row**, http://links.lww.com/CCM/H570). This finding persisted across all ED-triage groups with a time interval greater than 3.4 hours, except for the orange triage group, see **eTable 9**, **Models C–F** (http://links.lww.com/CCM/H570).

### Secondary Outcomes: ICU Mortality

After adjusting for APACHE-IV probability, no association with ICU mortality was observed in the entire cohort. After adjustment for ED triage, we found an ORs_adjED_ of 0.81 (95% CI, 0.72–0.92) for the 2.3–3.4 hours quintile and an ORs_adjED_ of 0.63 (95% CI, 0.55–0.72) for the quintile greater than 3.4 hours (**eTable 10**, http://links.lww.com/CCM/H570).

## DISCUSSION

This study aims to replicate prior findings and provide new insights into the relationship between ED-to-ICU time and hospital mortality. It extends previous research by analyzing subgroups in academic and NACT hospitals and examining how APACHE-IV probability and ED-triage scores modify this relationship.

In the entire cohort, the initial negative correlation between prolonged ED-to-ICU time and hospital mortality disappeared after adjusting for APACHE-IV probability. This suggests a potential link between these variables, consistent with previous studies (8–10, 23). Therefore, we further stratified patients by illness severity using APACHE-IV probability. A higher hospital mortality association was observed only in the group of patients where the APACHE-IV probability was greater than 55.4% and the ED-to-ICU time greater than 3.4 hours.

To determine if this effect is driven solely by patients in academic or NACT hospitals, subgroup analyses were conducted. In the academic cohort, a more pronounced association was observed, starting from an ED-to-ICU time greater than 1.1 hours. This aligns with our prior academic cohort study (8). Notably, this association was not observed in NACT hospitals, even among patients with the highest APACHE-IV probability.

This finding may be attributed to differences in disease severity between the two cohorts, with higher median APACHE-IV scores in academic hospitals. Initial diagnostic evaluations and stabilization occur in the ED in the Netherlands, followed by prompt transfer to the ICU for organ-support interventions. The patient-to-ICU nurse ratio is low, facilitating intensive treatment monitoring. ICU staff, specialized in complex organ support therapies, may initiate additional measures such as prone position mechanical ventilation and ECLS. High patient volumes in the ED may hinder the delivery of intensive care due to a higher patient-to-nurse ratio, compounded by limited specialized organ-support facilities. These factors may contribute to the observed association with higher hospital mortality in cases of prolonged ED-to-ICU times, particularly for patients in academic hospitals requiring this level of care. Therefore, the establishment of an ED-based ICU might hold promise as a potential solution for this subgroup (10). Due to the study’s retrospective nature and missing data (e.g., therapy adjustments and analysis of potential missed critical signals), this explanation remains theoretical. Prospective research with comprehensive registration is necessary to address this issue.

Patient characteristics and diagnosis may influence the association between ED-to-ICU time and hospital mortality. Groenland et al (8) demonstrated its significance for out-of-hospital cardiac arrest (OHCA) patients. Therefore, we performed a post hoc analysis focused on OHCA patients (**eTable 11**, http://links.lww.com/CCM/H570). In academic hospitals, prolonged ED-to-ICU time had a significant impact on hospital mortality, with durations greater than 3.4 hours resulting in an ORs_adjApache_ of 2.94 (95% CI, 1.80–4.78). This association was not evident in NACT hospitals and may be explained by the following.

First, academic hospitals receive the sickest patients, as indicated by the APACHE-IV probability. It is possible that these patients benefit more from early ICU bundle post-arrest care. Thus, prolonged ED LOS may be particularly harmful in academic hospitals. Second, according to the European Resuscitation Council guidelines (24), patients suspected of having ST-elevation myocardial infarction complicated by OHCA are directed to hospitals with PCI capabilities, frequently academic hospitals. Consequently, it is conceivable that patients who have undergone successful pre-hospital resuscitation without suspicion of high-risk occlusive coronary disease are more frequently admitted to NACT hospitals. Literature indicates that individuals with a shockable rhythm (which is more common in obstructive coronary disease) have faster return of spontaneous circulation (ROSC) and hence better outcomes (25, 26). In this context, the duration of ROSC may emerge as a more critical determinant of hospital mortality in NACT hospitals (27, 28) than the ED-to-ICU time.

Our analysis of ED-triage score adjustments and stratifications revealed a significant association with higher mortality rates exclusively among red-triaged patients admitted to an academic hospital. This association was found only for ED-to-ICU times of 1.6–3.4 hours and disappeared for patients ED-to-ICU times greater than 3.4 hours. It should be noted that the red-triaged group with greater than 3.4 hours comprises only 512 patients, leading to a less accurate estimate. A larger cohort is required to conclusively demonstrate the absence of this effect in this time frame. However, considering the evidence after adjusting for APACHE-IV probability and ED-triage, in our opinion, the association between prolonged ED-to-ICU time and increased mortality in academic hospitals appears to be strengthened.

In the NACT cohort, we observed a favorable association between ED-to-ICU time and mortality in the unadjusted data, which persisted after ED-triage score adjustment. Therefore, NACT EDs may have the resources to efficiently manage patients with relatively lower APACHE-IV scores, thereby reducing the need for rapid ICU admission.

Furthermore, the overall less clear and straightforward association between ED-to-ICU time and mortality after ED-triage score adjustment may be attributed to the limited adjustability of ED-triage scores, which primarily serve as rapid evaluation tool. For instance, the MTS categorizes patients into red (immediate attention), orange (very urgent), yellow (urgent), green (standard), and blue (nonurgent) based on specific criteria (29). However, patients within the same category may exhibit varying severity levels, despite sharing identical triage scores. This variability can lead to differences in waiting times within triage categories, potentially resulting in prolonged ED-to-ICU times for patients with high ED-triage scores but lower anticipated mortality rates. Adjustment and stratification based on ED-triage scores to assess the association between ED-to-ICU time and mortality appear less effective than hypothesized.

Our study has some limitations. First, its focus on Dutch hospitals may limit generalizability. Second, despite adjustments for disease severity using APACHE-IV probability and ED-triage scores, unaccounted confounding factors could influence the results. The partially missing ED-triage scores reduced the sample size, revealing higher APACHE-IV scores and predicted mortality rates, suggesting a sicker patient population. Including these patients might have increased the red/orange triage scores, potentially narrowing the CI for the observed association. Finally, some patients may have been misclassified as direct ED-to-ICU admissions, leading to inaccurate ED-to-ICU time assessments.

To further explore the ED-to-ICU time and mortality association, a scoring system using ED presentation data to predict ICU mortality upon admission would be interesting. This score would complement the triage score by providing a more precise evaluation of mortality risk rather than just assessing urgency. Candel et al (14) indicate that such an “APACHE on arrival” score yields higher predicted ICU mortality compared with the standard APACHE-IV probability.

## CONCLUSIONS

In patients with the highest APACHE-IV probability at academic hospitals, a prolonged ED-to-ICU time was associated with increased hospital mortality. We found no significant and consistent unfavorable association in lower APACHE-IV probability groups and in NACT hospitals. The association between longer ED-to-ICU time and higher mortality was not found after adjustment and stratification for ED-triage score. Further investigations are needed to explore the impact of ED-to-ICU time on specific admission diagnoses and to identify more appropriate adjustment and stratification factors.

## Supplementary Material
