# Supplementary material for: Emergency Department Triage, Transfer Times, and Hospital Mortality of Patients Admitted to the ICU: A Retrospective Replication and Continuation Study*
Source: Crit Care Med. 2024 Aug 19;52(12):1856–65. doi: 10.1097/CCM.0000000000006396 (PMC11556817; doi:10.1097/CCM.0000000000006396)
Supplement: Supplementary file 1 [file ccm-52-1856-s001.docx]

ONLINE SUPPLEMENTARY MATERIAL

The online supplementary material presented in this document are supplements to van Herwerden et al. with the title “ Emergency department triage, transfer times and hospital mortality of patients admitted to the Intensive Care Unit: a retrospective replication and continuation study”.

Inhoud

[eTable 1 Overview of Participating Hospitals with Patient Categories and Offered Therapies. 2](#_Toc166500698)

[eTable 2 baseline and in-hospital characteristics of patients with missing ED triage scores 3](#_Toc166500699)

[eTable 3 distribution of patient per ED-triage score and APACHE-IV probability group 4](#_Toc166500700)

[eTable 4 baseline and in-hospital characteristics in the academic hospital cohort 5](#_Toc166500701)

[eTable 5 baseline and in-hospital characteristics in the non-academic teaching hospital cohort 7](#_Toc166500702)

[eTable 6 odds ratios for hospital mortality in the overall cohort adjusted for APACHE-IV probability 9](#_Toc166500703)

[eTable 7 odds ratios for hospital mortality in the academic hospital cohort and non-academic teaching hospital cohort adjusted for APACHE-IV probability 11](#_Toc166500704)

[eTable 8 odds ratios for hospital mortality in the overall cohort adjusted for ED triage 13](#_Toc166500705)

[eTable 9 odds ratios for hospital mortality in the academic hospital cohort and non-academic teaching hospital cohort adjusted for ED triage 15](#_Toc166500706)

[eTable 10 odds ratios for ICU mortality in the overall cohort adjusted for APACHE-IV probability and ED triage 17](#_Toc166500707)

[eTable 11 odds ratios for hospital mortality in the overall, academic hospital cohort and non-academic teaching hospital cohort of patients treated for an cardiac arrest adjusted for APACHE III 19](#_Toc166500708)

[eFigure 1 odds ratios for hospital mortality per length of stay in the emergency department, for each Acute Physiology and Chronic Health Evaluation quintile 21](#_Toc166500709)

[eFigure 2 odds ratios for hospital mortality per length of stay in the emergency department, for each ED triage score 22](#_Toc166500710)

# eTable 1 Overview of Participating Hospitals with Patient Categories and Offered Therapies.

|  | Academic | Teaching | MV | RRT | ECMO | PCI | TC | NC | ALT | BT | RTx | LTx | HTx | LuTx |
| --- | --- | --- | --- | --- | --- | --- | --- | --- | --- | --- | --- | --- | --- | --- |
| Erasmus University Medical Center | X |  | X | X | X | X | X | X | X |  | X | X | X | X |
| Radboud University Medical Center | X |  | X | X | X | X | X | X | X |  | X |  |  |  |
| Leiden University Medical Center | X |  | X | X | X | X | X | X | X |  | X | X |  |  |
| University Medical Center Groningen | X |  | X | X | X | X | X | X | X |  | X | X | X | X |
| Gelre Hospitals |  | X | X | X |  |  |  |  |  |  |  |  |  |  |
| Haaglanden Medisch Centrum |  | X | X | X |  | X | X | X |  |  |  |  |  |  |
| Ikazia Hospital |  | X | X | X |  |  |  |  |  |  |  |  |  |  |
| Maasstad Hospital |  | X | X | X |  | X |  |  |  | X |  |  |  |  |
| Medisch Spectrum Twente |  | X | X | X | X | X | X | X | X |  |  |  |  |  |
| Reinier de Graaf Gasthuis |  | X | X | X |  |  |  |  | X |  |  |  |  |  |
| Treant Zorggroep |  | X | X | X |  | X |  |  |  |  |  |  |  |  |
| Zuyderland Medisch Centrum |  | X | X | X |  | X |  |  | X |  |  |  |  |  |

MV=Mechanical Ventilation, RRT=Renal Replacement Therapy, ECMO=Extracorporeal Membrane Oxygenation, PCI=Percutaneous Coronary Intervention, TC=Trauma center, NC=Neurosurgery center, ALT= Centers capable of providing (partially) acute leukemia treatment., BT=Burn patient treatment center, RTx=Renal Transplantation center, LTx= Liver Transplantation center, HTx=Heart Transplantation center, LuTx= Lunt Transplantation center.

# eTable 2 baseline and in-hospital characteristics of patients with missing ED triage scores

| **Baseline characteristics** | All Patients  n=6,387 | ED to ICU time < 1.1 hr  n=1,632 | ED to ICU time 1.1-1.6 hr  n=1,284 | ED to ICU time 1.6-2.3 hr  n=1,364 | ED to ICU time 2.3-3.4 hr  n=1,177 | ED to ICU time >3.4 hr  n=930 | p-value |
| --- | --- | --- | --- | --- | --- | --- | --- |
| Age, yr, median [IQR] | 60 [44-72] | 61 [46-72] | 58 [41-70] | 58 [41-70] | 60 [45-71] | 64 [50-74] | <0.001 |
| Male, gender, n (%) | 3,856 (60.4) | 999 (61.2) | 791 (61.6) | 830 (60.9) | 711 (60.4) | 525 (56.5) | 0.116 |
| APACHE-IV score, median [IQR] | 66 [42-94] | 76 [51-105] | 67 [42-98] | 65 [42-95] | 58 [37-85] | 58 [39-80] | <0.001 |
| APACHE-IV predicted mortality, median [IQR] | 0.17 [0.04-0.51] | 0.28 [0.08-0.67] | 0.19 [0.04-0.60] | 0.18 [0.04-0.52] | 0.11 [0.03-0.37] | 0.11 [0.04-0.27] | <0.001 |
|  |  |  |  |  |  |  |  |
| **Hospital type** |  |  |  |  |  |  |  |
| *Academic* | 1,548 (24.2) | 273 (16.7) | 317 (24.7) | 388 (28.4) | 340 (19.1) | 230 (24.7) |  |
| *Non-academic* | 4,839 (75.8) | 1,359 (83.3) | 967 (75.3) | 976 (71.6) | 837 (80.9) | 700 (75.3) |  |
|  |  |  |  |  |  |  |  |
| **In-hospital characteristics** |  |  |  |  |  |  |  |
| ED to ICU time, hr, median [IQR] | 1.7 [1.1-2.7] | 0.8 [0.6-0.9] | 1.3 [1.2-1.5] | 1.9 [1.7-2.1] | 2.7 [2.5-3.1] | 4.4 [3.8-5.5] | <0.001 |
| ICU LOS, d, median [IQR] | 1.8 [0.8-4.5] | 1.9 [0.8-4.8] | 1.9 [0.8-5.1] | 1.8 [0.8-4.8] | 1.5 [0.7-3.8] | 1.6 [0.8-3.7] | <0.001 |
| Hospital LOS, d, median [IQR] | 7.9 [3.0-15.6] | 7.0 [2.0-16.0] | 7.0 [2.6-15.2] | 8.00 [3.0-15.0] | 8.0 [3.0-15.1] | 8.5 [4.0-15.0] | <0.001 |
| ICU mortality, n (%) | 1,162 (18.2) | 392 (24.0) | 250 (19.5) | 247 (18.1) | 168 (14.3) | 105 (11.3) | <0.001 |
| Hospital mortality, n (%) | 1,360 (21.3) | 436 (26.7) | 289 (22.5) | 294 (21.6) | 200 (17.0) | 141 (15.2) | <0.001 |

APACHE = Acute Physiology and Chronic Health Evaluation, ED to ICU time = emergency department to intensive care unit time, IQR = interquartile range, LOS = length of stay

# eTable 3 distribution of patient per ED-triage score and APACHE-IV probability group

|  | ED-triage score  - *Red*  N=8,623, (%) | ED-triage score  - *Orange*  N=10,013, (%) | ED-triage score  - *Yellow*  N=2,938, (%) | ED-triage score  - *Green & Blue*  N=494, (%) |
| --- | --- | --- | --- | --- |
| APACHE-IV probability  <8.0% | 1,891 (21.9) | 5,164 (51.6) | 1,804 (61.4) | 289 (58.5) |
| APACHE-IV probability 8.0%- 20.4 % | 1,470 (17.0) | 2,336 (23.3) | 581 (19.8) | 97 (19.6) |
| APACHE-IV probability 20.4%-55.4% | 2,031 (23.6) | 1,757 (17.5) | 376 (12.8) | 66 (13.4) |
| APACHE-IV probability  >55.4 % | 3,231 (37.5) | 756 (7.6) | 177 (6.0) | 42 (8.5) |

X-squared = 4138.9, df = 9, p-value < 0.001

# eTable 4 baseline and in-hospital characteristics in the academic hospital cohort

| **Baseline characteristics** | All Patients  n=10,279 | ED to ICU time  < 1.1 hr  n=1,307 | ED to ICU time 1.1-1.6 hr  n=2,057 | ED to ICU time 1.6-2.3 hr  n=2,325 | ED to ICU time 2.3-3.4 hr  n=2,121 | ED to ICU time  >3.4 hr  n=2,469 | p-value |
| --- | --- | --- | --- | --- | --- | --- | --- |
| Age, yr, median [IQR] | 61 [46-72] | 62 [46-73] | 60 [44-72] | 60 [45-71] | 61 [46-72] | 61 [48-71] | <0.001 |
| Male, gender, n (%) | 6,341 (61.7) | 815 (62.4) | 1,314 (63.9) | 1,472 (63.3) | 1,299 (61.2) | 1,441 (58.4) | 0.002 |
| APACHE-IV score, median [IQR] | 66 [43 – 95] | 78 [51 – 105] | 74 [48 – 102] | 72 [47 – 104] | 65 [41 – 95] | 53 [36 – 76] | <0.001 |
| APACHE-IV predicted mortality, median [IQR] | 0.19 [0.05-0.56] | 0.33 [0.10-0.70] | 0.26 [0.08–0.66] | 0.26 [0.07–0.66] | 0.17 [0.05–0.52] | 0.10 [0.03–0.26] | <0.001 |
|  |  |  |  |  |  |  |  |
| ED-triage score available, n (%) | 8,731 (84.9) | 1,032 (79.0) | 1,740 (84.6) | 1,937 (83.3) | 1,781 (84.0) | 2,239 (90.7) |  |
| ED-triage score missing, n (%) | 1,548 (15.1) | 275 (21.0) | 317 (15.4) | 388 (16.7) | 340 (16.0) | 230 (9.3) |  |
|  |  |  |  |  |  |  |  |
| **ED-triage score *^a^*** |  |  |  |  |  |  |  |
| *Red* | 4,592 (52.5) | 755 (73.1) | 1,248 (71.7) | 1,246 (64.0) | 831 (46.6) | 512 (22.9) | <0.001 |
| *Orange* | 3,196 (36.6) | 258 (25.0) | 446 (25.6) | 595 (30.6) | 744 (41.7) | 1,153 (51.5) | <0.001 |
| *Yellow* | 836 (9.6) | 18 (1.7) | 39 (2.2) | 89 (4.6) | 194 (10.9) | 496 (22.2) | <0.001 |
| *Green & Blue* | 107 (1.2) | 1 (0.1) | 7 (0.4) | 7 (0.4) | 12 (0.7) | 78 (3.5) | <0.001 |
|  |  |  |  |  |  |  |  |
|  |  |  |  |  |  |  |  |
| **Most common admission diagnoses, n (%) *^b^*** |  |  |  |  |  |  |  |
|  |  |  |  |  |  |  |  |
| Cardiac Arrest | 1,872 (18.2) | 389 (29.8) | 458 (22.3) | 557 (24.0) | 358 (16.9) | 110 (4.5) | <0.01 |
| Trauma (non-operative) | 1,739 (16.9) | 150 (11.5) | 550 (26.7) | 767 (33.0) | 625 (29.5) | 540 (21.9) | <0.01 |
| Intracranial/subdural/epidural haemorrhage | 841 (8.2) | 134 (10.3) | 216 (10.5) | 228 (9.8) | 149 (7.0) | 114 (4.6) | <0.01 |
| Respiratory failure | 923 (9.0) | 98 (7.5) | 158 (7.7) | 199 (8.6) | 201 (9.5) | 267 (10.8) | <0.01 |
| Overdose | 607 (5.9) | 82 (6.3) | 129 (6.3) | 135 (5.8) | 145 (6.8) | 116 (4.7) | 0.03 |
| Sepsis | 474 (4.6) | 16 (1.2) | 51 (2.5) | 82 (3.5) | 131 (6.2) | 194 (7.9) | <0.01 |
| Pneumonia | 72 (0.7) | 131 (10.0) | 131 (6.4) | 181 (7.8) | 224 (10.6) | 739 (29.9) | <0.01 |
| Trauma (operative) | 244 (2.4) | 18 (1.4) | 33 (1.6) | 49 (2.1) | 60 (2.8) | 84 (3.4) | <0.01 |
| Acute coronary syndrome | 223 (2.2) | 28 (2.1) | 27 (1.3) | 47 (2.0) | 48 (2.3) | 73 (3.0) | <0.01 |
| Aneurysm | 148 (1.4) | 18 (1.4) | 27 (1.3) | 25 (1.1) | 26 (1.2) | 52 (2.1) | 0.02 |

| eTable 4 baseline and in-hospital characteristics in the academic hospital cohort | | | | | | | |
| --- | --- | --- | --- | --- | --- | --- | --- |
| **In-hospital characteristics** |  |  |  |  |  |  |  |
| ED to ICU time, hr, median [IQR] | 2.1 [1.4-3.4] | 0.9 [0.7 – 1.0] | 1.3 [1.2 – 4.3] | 1.9 [1.7 – 2.1] | 2.8 [2.5 – 3.1] | 4.7 [4.0 – 6.0] | <0.001 |
| ICU LOS, d, median ([QR] | 1.7 [0.7-4.1] | 1.8 [0.7 – 4.5] | 1.8 [0.8 – 4.3] | 1.8 [0.8 – 4.7] | 1.7 [0.7 – 4.2] | 1.5 [0.7 – 3.4] | <0.001 |
| Hospital LOS, d, median [IQR] | 6.0 [2.0-13.7] | 4.5 [1.5 –3.0] | 5.0 [1.8 – 2.9] | 6.0 [2.0 - 3.0] | 6.0 [2.0 – 13.6] | 7.1 [3.0 – 5.0] | 0.005 |
| ICU mortality, n (%) | 2,056 (20.0) | 312 (23.9) | 504 (24.5) | 557 (24.0) | 408 (19.2) | 275 (11.1) | <0.001 |
| Hospital mortality, n (%) | 2,424 (23.6) | 352 (26.9) | 574 (27.9) | 638 (27.4) | 477 (22.5) | 383 (15.5) | <0.001 |

APACHE = Acute Physiology and Chronic Health Evaluation, ED to ICU time = emergency department to ICU time, IQR = interquartile range, LOS = length of stay

*a* Percentages were calculated based on admissions where an ED-triage score was available (n=8,731).

*b* Only the ten most reported diagnose groups are reported.

# eTable 5 baseline and in-hospital characteristics in the non-academic teaching hospital cohort

| **Baseline characteristics** | All Patients  n=18,176 | ED to ICU time  < 1.1 hr  n=4,519 | ED to ICU time 1.1-1.6 hr  n=3,383 | ED to ICU time 1.6-2.3 hr  n=3,486 | ED to ICU time 2.3-3.4 hr  n= 3,590 | ED to ICU time  >3.4 hr  n=3,198 | p-value |
| --- | --- | --- | --- | --- | --- | --- | --- |
| Age, yr, median [IQR] | 61 [47-73] | 61 [46-72] | 60 [72-112] | 60 [45-72] | 61 [47-73] | 64 [51-75] | <0.001 |
| Male, gender, n (%) | 10,411 (57.3) | 2,598 (57.5) | 1,940 (57.3) | 2,040 (58.5) | 2,042(56.9) | 1791 (56.0) | 0.32 |
| APACHE-IV score, median [IQR] | 57 [36-85] | 68 [43-98] | 62 [39-92] | 57 [35-85] | 51 [33-75] | 49 [34-71] | <0.001 |
| APACHE-IV predicted mortality, median [IQR] | 0.10 [0.03-0.35] | 0.17 [0.04-0.58] | 0.13 [0.03-0.46] | 0.10 [0.02-0.36] | 0.07 [0.02-0.22] | 0.07 [0.02-0.19] | <0.001 |
|  |  |  |  |  |  |  |  |
| ED-triage score available, n (%) | 13,337 (73.4) | 3,160 (69.9) | 2,416 (71.4) | 2,510 (72.0) | 2,753 (76.7) | 2,498 (78.1) |  |
| ED-triage score missing, n (%) | 4,839 (26.6) | 1,359 (30.1) | 967 (28.6) | 976 (28.0) | 837 (23.3) | 700 (21.9) |  |
|  |  |  |  |  |  |  |  |
| **ED-triage score *^a^*** |  |  |  |  |  |  |  |
| *Red* | 4,031 (30.2) | 1,652 (52.3) | 938 (38.8) | 711 (28.3) | 491 (17.8) | 239 (9.6) | <0.001 |
| *Orange* | 6,817 (51.1) | 1,207 (38.2) | 1,193 (49.3) | 1,367 (54.4) | 1,608 (58.4) | 1,442 (57.7) | <0.001 |
| *Yellow* | 2,102 (15.8) | 234 (7.4) | 219 (9.1) | 372 (14.8) | 569 (20.7) | 708 (28.3) | <0.001 |
| *Green & Blue* | 387 (2.9) | 67 (2.1) | 66 (2.7) | 60 (2.4) | 85 (3.1) | 109 (4.4) | <0.001 |
|  |  |  |  |  |  |  |  |
|  |  |  |  |  |  |  |  |
| **Most common admission diagnoses, n (%) *^b^*** |  |  |  |  |  |  |  |
|  |  |  |  |  |  |  |  |
| Cardiac Arrest | 1,946 (10.7) | 809 (17.9) | 511 (15.1) | 404 (11.6) | 188 (5.2) | 34 (1.1) | <0.01 |
| Trauma (non-operative) | 2,048 (11.3) | 347 (7.7) | 336 (9.9) | 348 (10.0) | 495 (13.8) | 522 (16.3) | <0.01 |
| Intracranial/subdural/epidural haemorrhage | 789 (4.3) | 299 (6.6) | 147 (4.3) | 142 (4.1) | 106 (3.0) | 95 (3.0) | <0.01 |
| Respiratory failure | 1,764 (9.7) | 516 (11.4) | 325 (9.6) | 305 (8.7) | 324 (9.0) | 294 (9.2) | <0.01 |
| Overdose | 2,521 (13.9) | 673 (14.9) | 545 (16.1) | 573 (16.4) | 476 (13.3) | 254 (7.9) | <0.01 |
| Sepsis | 1,453 (8.0) | 214 (4.7) | 207 (6.1) | 277 (7.9) | 379 (10.6) | 376 (11.8) | <0.01 |
| Pneumonia | 1,640 (0.9) | 426 (9.4) | 308 (9.1) | 325 (9.3) | 344 (9.6) | 237 (7.4) | 0.01 |
| Trauma (operative) | 138 (0.8) | 22 (0.5) | 26 (0.8) | 24 (0.7) | 40 (1.1) | 26 (0.8) | 0.02 |
| Acute coronary syndrome | 168 (0.9) | 44 (1.0) | 34 (1.0) | 38 (1.1) | 28 (0.8) | 24 (0.8) | 0.09 |
| Aneurysm | 177 (1.0) | 40 (0.9) | 24 (0.7) | 34 (1.0) | 29 (0.8) | 50 (1.6) | 0.27 |

| eTable 5 baseline and in-hospital characteristics in the non-academic teaching hospital cohort | | | | | | | |
| --- | --- | --- | --- | --- | --- | --- | --- |
| **In-hospital characteristics** |  |  |  |  |  |  |  |
| ED to ICU time, hr, median [IQR] | 1.8 [1.1-2.9] | 0.7 [0.5-0.9] | 1.3 [1.2-1.6] | 1.9 [1.7-2.1] | 2.8 [2.5–3.1] | 4.5 [3.9-5.6] | <0.001 |
| ICU LOS, d, median [IQR] | 1.4 [0.7-3.2] | 1.6 [0.7-3.8] | 1.5 [0.7-3.8] | 1.4 [0.7-3.3] | 1.3 [0.7-2.9] | 1.3 [0.7-2.6] | <0.001 |
| Hospital LOS, d, median [IQR] | 7.0 [3.0-14.0] | 6.0 [2.0-14.0] | 7.0 [2.0-14.0] | 7.0 [2.5-13.0] | 7.0 [3.0-14.0] | 7.6 [4.0-14.0] | 0.09 |
| ICU mortality, n (%) | 2,371 (13.0) | 884 (19.6) | 513 (15.2) | 445 (12.8) | 313 (8.7) | 216 (6.8) | <0.001 |
| Hospital mortality, n (%) | 3,114 (17.1) | 1,085 (24) | 644 (19.0) | 595 (17.1) | 446 (12.4) | 344 (10.8) | <0.001 |

APACHE = Acute Physiology and Chronic Health Evaluation, ED to ICU time = emergency department to ICU time, IQR = interquartile range, LOS = length of stay

*a* Percentages were calculated based on admissions where an ED-triage score was available (n=8,731).

*b* Only the ten most reported diagnose groups are reported.

# eTable 6 odds ratios for hospital mortality in the overall cohort adjusted for APACHE-IV probability

| **Model *^a^*** | **Hospital mortality** | **p-value** |
| --- | --- | --- |
| ‘’A’’ ED to ICU time; |  |  |
| ED to ICU time <1.1 hr | Reference | p<0.01 |
| ED to ICU time 1.1-1.6 hr | 0.84 (0.77-0.91) *b* |  |
| ED to ICU time 1.6-2.3 hr | 0.77 (0.71-0.84) *b* |  |
| ED to ICU time 2.3-3.4 hr | 0.56 (0.51-0.61) *b* |  |
| ED to ICU time >3.4 hr | 0.41 (0.37-0.45) *b* |  |
|  |  |  |
| ‘’B’’ ED to ICU time; APACHE IV probability |  |  |
| ED to ICU time <1.1 hr | Reference | p=0.36 |
| ED to ICU time 1.1-1.6 hr | 0.93 (0.84-1.04) |  |
| ED to ICU time 1.6-2.3 hr | 0.98 (0.88-1.09) |  |
| ED to ICU time 2.3-3.4 hr | 0.98 (0.88-1.10) |  |
| ED to ICU time >3.4 hr | 1.06 (0.94-1.19) |  |
|  |  |  |
| ‘’C’’ ED to ICU time x APACHE < 8.0% |  |  |
| ED to ICU time <1.1 hr | Reference | p=0.87 |
| ED to ICU time 1.1-1.6 hr | 1.06 (0.62-1.80) |  |
| ED to ICU time 1.6-2.3 hr | 1.22 (0.74-2.01) |  |
| ED to ICU time 2.3-3.4 hr | 0.96 (0.58-1.58) |  |
| ED to ICU time >3.4 hr | 1.06 (0.65-1.72) |  |
|  |  |  |
| ‘’D’’ ED to ICU time x APACHE 8.0%- 20.4  % |  |  |
| ED to ICU time <1.1 hr | Reference | p=0.40 |
| ED to ICU time 1.1-1.6 hr | 1.00 (0.73-1.35) |  |
| ED to ICU time 1.6-2.3 hr | 1.01 (0.75-1.37) |  |
| ED to ICU time 2.3-3.4 hr | 1.00 (0.75-1.35) |  |
| ED to ICU time >3.4 hr | 0.80 (0.59-1.08) |  |
|  |  |  |
| ‘’E’’ ED to ICU time x APACHE 20.4%- 55.4% |  |  |
| ED to ICU time <1.1 hr | Reference | p=0.40 |
| ED to ICU time 1.1-1.6 hr | 0.92 (0.76-1.10) |  |
| ED to ICU time 1.6-2.3 hr | 0.94 (0.78-1.13) |  |
| eTable 6 odds ratios for hospital mortality in the overall cohort adjusted for APACHE-IV probability | | |
| ED to ICU time 2.3-3.4 hr | 0.96 (0.79-1.16) |  |
| ED to ICU time >3.4 hr | 1.09 (0.92-1.32) |  |
|  |  |  |
| ‘’F’’ ED to ICU time x APACHE >55.4 % |  |  |
| ED to ICU time <1.1 hr | Reference | p=0.12 |
| ED to ICU time 1.1-1.6 hr | 0.92 (0.79-1.06) |  |
| ED to ICU time 1.6-2.3 hr | 0.98 (0.84-1.13) |  |
| ED to ICU time 2.3-3.4 hr | 0.99 (0.78-1.05) |  |
| ED to ICU time >3.4 hr | 1.24 (1.00-1.54) *b* |  |

APACHE = Acute Physiology and Chronic Health Evaluation, ED to ICU time = emergency department to intensive care unit time.

*^a^* All models are adjusted for hospital

*b* p < 0.05.

Values represent the odds ratios and 95% CIs.

The p is analyzing whether ED to ICU time as a total factor is associated with the hospital mortality, we used a Wald test for the ED to ICU variables

# eTable 7 odds ratios for hospital mortality in the academic hospital cohort and non-academic teaching hospital cohort adjusted for APACHE-IV probability

| **Model Academic cohort *^a^*** | **Hospital mortality** | **p** | **Model non-academic cohort *^a^*** | **Hospital mortality** | p-value |
| --- | --- | --- | --- | --- | --- |
| ‘’A’’ ED to ICU time; |  |  | ‘’A’’ ED to ICU time; |  |  |
| ED to ICU time <1.1 hr | Reference | p<0.01 | ED to ICU time <1.1 hr | Reference | p<0.01 |
| ED to ICU time 1.1-1.6 hr | 1.07 (0.92-1.25) |  | ED to ICU time 1.1-1.6 hr | 0.75 (0.68-0.84) *b* |  |
| ED to ICU time 1.6-2.3 hr | 1.04 (0.89-1.21) |  | ED to ICU time 1.6-2.3 hr | 0.66 (0.59-0.74) *b* |  |
| ED to ICU time 2.3-3.4 hr | 0.80 (0.68-0.94) *b* |  | ED to ICU time 2.3-3.4 hr | 0.45 (0.40-0.51) *b* |  |
| ED to ICU time >3.4 hr | 0.51 (0.43-0.60) *b* |  | ED to ICU time >3.4 hr | 0.38 (0.33-0.43) *b* |  |
|  |  |  |  |  |  |
| ‘’B1’’ ED to ICU time; APACHE-IV  probability |  |  | ‘’B2’’ ED to ICU time; APACHE-IV probability |  |  |
| ED to ICU time <1.1 hr | Reference | p=0.08 | ED to ICU time <1.1 hr | Reference | p=0.07 |
| ED to ICU time 1.1-1.6 hr | 1.18 (0.98-1.42) |  | ED to ICU time 1.1-1.6 hr | 0.83 (0.72-0.95) *b* |  |
| ED to ICU time 1.6-2.3 hr | 1.21 (1.01-1.46) *b* |  | ED to ICU time 1.6-2.3 hr | 0.88 (0.77-1.01) |  |
| ED to ICU time 2.3-3.4 hr | 1.21 (1.00-1.46) *b* |  | ED to ICU time 2.3-3.4 hr | 0.88 (0.76-1.02) |  |
| ED to ICU time >3.4 hr | 1.34 (1.10-1.64) *b* |  | ED to ICU time >3.4 hr | 0.92 (0.79-1.08) |  |
|  |  |  |  |  |  |
| ‘’C1’’ ED to ICU time x APACHE < 8.0% |  |  | ‘’C2’’ ED to ICU time x APACHE < 8.0% |  |  |
| ED to ICU time <1.1 hr | Reference | p=0.34 | ED to ICU time <1.1 hr | Reference | p=0.59 |
| ED to ICU time 1.1-1.6 hr | 0.73 (0.29-1.88) |  | ED to ICU time 1.1-1.6 hr | 1.18 (0.58-2.14) |  |
| ED to ICU time 1.6-2.3 hr | 1.03 (0.44-2.40) |  | ED to ICU time 1.6-2.3 hr | 1.11 (0.60-2.08) |  |
| ED to ICU time 2.3-3.4 hr | 0.89 (0.38-2.07) |  | ED to ICU time 2.3-3.4 hr | 0.80 (0.42-1.54) |  |
| ED to ICU time >3.4 hr | 0.53 (0.22-1.25) |  | ED to ICU time >3.4 hr | 1.32 (0.74-2.40) |  |
|  |  |  |  |  |  |
| ‘’D1’’ ED to ICU time x APACHE 8.0%-  20.4 % |  |  | ‘’D2’’ ED to ICU time x APACHE 8.0%- 20.4 % |  |  |
| ED to ICU time <1.1 hr | Reference | p=0.17 | ED to ICU time <1.1 hr | Reference | p<0.01 |
| ED to ICU time 1.1-1.6 hr | 1.34 (0.71-2.53) |  | ED to ICU time 1.1-1.6 hr | 0.94 (0.66-1.35) |  |
| ED to ICU time 1.6-2.3 hr | 0.92 (0.47-1.80) |  | ED to ICU time 1.6-2.3 hr | 1.15 (0.81-1.61) |  |
| ED to ICU time 2.3-3.4 hr | 1.49 (0.80-2.79) |  | ED to ICU time 2.3-3.4 hr | 0.90 (0.63-1.27) |  |
| ED to ICU time >3.4 hr | 1.59 (0.88-2.87) |  | ED to ICU time >3.4 hr | 0.50 (0.33-0.76) *b* |  |
|  |  |  |  |  |  |
| ‘’E1’’ ED to ICU time x APACHE 20.4%- 55.4% |  |  | ‘’E2’’ ED to ICU time x APACHE 20.4%-55.4% |  |  |
| ED to ICU time <1.1 hr | Reference | p=0.81 | ED to ICU time <1.1 hr | Reference | P=0.33 |
| ED to ICU time 1.1-1.6 hr | 1.02 (0.74-1.42) |  | ED to ICU time 1.1-1.6 hr | 0.84 (0.66-1.06) |  |
| ED to ICU time 1.6-2.3 hr | 1.00 (0.73-1.39) |  | ED to ICU time 1.6-2.3 hr | 0.89 (0.70-1.13) |  |

| eTable 7 odds ratios for hospital mortality in the academic hospital cohort and non-academic teaching hospital cohort adjusted for APACHE-IV probability | | | | | |
| --- | --- | --- | --- | --- | --- |
| ED to ICU time 2.3-3.4 hr | 0.93 (0.67-1.30) |  | ED to ICU time 2.3-3.4 hr | 0.99 (0.78-1.26) |  |
| ED to ICU time >3.4 hr | 1.12 (0.80-1.55) |  | ED to ICU time >3.4 hr | 1.08 (0.85-1.38) |  |
|  |  |  |  |  |  |
| ‘’F1’’ ED to ICU time x APACHE >55.4 % |  |  | ‘’F2’’ ED to ICU time x APACHE >55.4 % |  |  |
| ED to ICU time <1.1 hr | Reference | p=0.02 | ED to ICU time <1.1 hr | Reference | p=0.02 |
| ED to ICU time 1.1-1.6 hr | 1.27 (1.00-1.63) *b* |  | ED to ICU time 1.1-1.6 hr | 0.77 (0.64-0.94) *b* |  |
| ED to ICU time 1.6-2.3 hr | 1.40 (1.09-1.78) *b* |  | ED to ICU time 1.6-2.3 hr | 0.78 (0.64-0.95) *b* |  |
| ED to ICU time 2.3-3.4 hr | 1.35 (1.04-1.76) *b* |  | ED to ICU time 2.3-3.4 hr | 0.82 (0.64-1.04) |  |
| ED to ICU time >3.4 hr | 1.64 (1.19-2.25) *b* |  | ED to ICU time >3.4 hr | 1.08 (0.79-1.47) |  |

APACHE = Acute Physiology and Chronic Health Evaluation, ED to ICU time = emergency department to intensive care unit time

*a* All models are adjusted for hospital

*b* p < 0.05.

Values represent the odds ratios and 95% CIs.

The p is analyzing whether ED to ICU time as a total factor is associated with the hospital, we used a Wald test for the ED to ICU variables

# eTable 8 odds ratios for hospital mortality in the overall cohort adjusted for ED triage

| **Model *^a^*** | **Hospital mortality** | **p-value** |
| --- | --- | --- |
| ‘’A’’ ED to ICU time |  |  |
| ED to ICU time <1.1 hr | Reference | p<0.01 |
| ED to ICU time 1.1-1.6 hr | 0.84 (0.77-0.91) |  |
| ED to ICU time 1.6-2.3 hr | 0.77 (0.71-0.84) |  |
| ED to ICU time 2.3-3.4 hr | 0.56 (0.51-0.61) |  |
| ED to ICU time >3.4 hr | 0.41 (0.37-0.45) |  |
|  |  |  |
| ‘’B’’ ED to ICU time; Triage score |  |  |
| ED to ICU time <1.1 hr | Reference | p<0.01 |
| ED to ICU time 1.1-1.6 hr | 0.94 (0.85-1.05) |  |
| ED to ICU time 1.6-2.3 hr | 0.96 (0.87-1.07) |  |
| ED to ICU time 2.3-3.4 hr | 0.82 (0.73-0.91) *b* |  |
| ED to ICU time >3.4 hr | 0.73 (0.64-0.82) *b* |  |
|  |  |  |
| ‘’C’’ ED to ICU time x Triage score 4 (blue/ green) |  |  |
| ED to ICU time <1.1 hr | Reference | p<0.01 |
| ED to ICU time 1.1-1.6 hr | 0.90 (0.75-1.08) |  |
| ED to ICU time 1.6-2.3 hr | 0.89 (0.75-1.05) |  |
| ED to ICU time 2.3-3.4 hr | 0.75 (0.64-0.88) *b* |  |
| ED to ICU time >3.4 hr | 0.71 (0.60-0.82) *b* |  |
|  |  |  |
| ‘’D’’ ED to ICU time x Triage score 3 (yellow) |  |  |
| ED to ICU time <1.1 hr | Reference | p<0.01 |
| ED to ICU time 1.1-1.6 hr | 0.73 (0.44-1.18) |  |
| ED to ICU time 1.6-2.3 hr | 0.69 (0.45-1.04) |  |
| ED to ICU time 2.3-3.4 hr | 0.45 (0.30-0.67) *b* |  |
| ED to ICU time >3.4 hr | 0.54 (0.38-0.77) *b* |  |
|  |  |  |
| ‘’E’’ ED to ICU time x Triage score 2 (orange) |  |  |
| ED to ICU time <1.1 hr | Reference | p=0.01 |
| ED to ICU time 1.1-1.6 hr | 0.95 (0.77-1.16) |  |
| ED to ICU time 1.6-2.3 hr | 0.89 (0.73-1.08) |  |

| eTable 8 odds ratios for hospital mortality in the overall cohort adjusted for ED triage | | |
| --- | --- | --- |
| ED to ICU time 2.3-3.4 hr | 0.91 (0.76-1.10) |  |
| ED to ICU time >3.4 hr | 0.79 (0.66-0.96) *b* |  |
|  |  |  |
| ‘’F’’ ED to ICU time x Triage score 1 (red) |  |  |
| ED to ICU time <1.1 hr | Reference | p<0.01 |
| ED to ICU time 1.1-1.6 hr | 1.05 (0.92-1.20) |  |
| ED to ICU time 1.6-2.3 hr | 1.15 (0.99-1.31) |  |
| ED to ICU time 2.3-3.4 hr | 1.03 (0.87-1.21) |  |
| ED to ICU time >3.4 hr | 0.82 (0.65-1.03) |  |

ED to ICU time = emergency department to intensive care unit time.

*^a^* All models are adjusted for hospital

*b* p < 0.05.

Values represent the odds ratios and 95% CIs.

The p is analyzing whether ED to ICU time as a total factor is associated with the hospital mortality, we used a Wald test for the ED to ICU variables

# eTable 9 odds ratios for hospital mortality in the academic hospital cohort and non-academic teaching hospital cohort adjusted for ED triage

| **Model *^a^*** | **Hospital mortality** | **p** | **Model *^a^*** | **Hospital mortality** | p-value |
| --- | --- | --- | --- | --- | --- |
| ‘’A’’ ED to ICU time |  |  | ‘’A’’ ED to ICU time; |  |  |
| ED to ICU time <1.1 hr | Reference | p<0.01 | ED to ICU time <1.1 hr | Reference | p<0.01 |
| ED to ICU time 1.1-1.6 hr | 1.07 (0.92-1.25) |  | ED to ICU time 1.1-1.6 hr | 0.75 (0.68-0.84) *b* |  |
| ED to ICU time 1.6-2.3 hr | 1.04 (0.89-1.21) |  | ED to ICU time 1.6-2.3 hr | 0.66 (0.59-0.74) *b* |  |
| ED to ICU time 2.3-3.4 hr | 0.80 (0.68-0.94) *b* |  | ED to ICU time 2.3-3.4 hr | 0.45 (0.40-0.51) *b* |  |
| ED to ICU time >3.4 hr | 0.51 (0.43-0.60) *b* |  | ED to ICU time >3.4 hr | 0.38 (0.33-0.43) *b* |  |
|  |  |  |  |  |  |
| ‘’B1’’ ED to ICU time; Triage score |  |  | ‘’B2’’ ED to ICU time; and Triage score |  |  |
| ED to ICU time <1.1 hr | Reference | p=0.08 | ED to ICU time <1.1 hr | Reference | p<0.01 |
| ED to ICU time 1.1-1.6 hr | 1.13 (0.95-1.36) |  | ED to ICU time 1.1-1.6 hr | 0.87 (0.76-1.00) *b* |  |
| ED to ICU time 1.6-2.3 hr | 1.21 (1.01-1.44) |  | ED to ICU time 1.6-2.3 hr | 0.86 (0.75-0.99) *b* |  |
| ED to ICU time 2.3-3.4 hr | 1.09 (0.91-1.32) |  | ED to ICU time 2.3-3.4 hr | 0.71 (0.61-0.82) *b* |  |
| ED to ICU time >3.4 hr | 0.98 (0.81-1.19) |  | ED to ICU time >3.4 hr | 0.64 (0.54-0.75) *b* |  |
|  |  |  |  |  |  |
| ‘’C1’’ ED to ICU time x Triage score 4 (blue/green) |  |  | ‘’C2’’ ED to ICU time x Triage score 4 (blue/green) |  |  |
| ED to ICU time <1.1 hr | Reference | p<0.01 | ED to ICU time <1.1 hr | Reference | p<0.01 |
| ED to ICU time 1.1-1.6 hr | 0.91 (0.59-1.41) |  | ED to ICU time 1.1-1.6 hr | 0.88 (0.71-1.08) |  |
| ED to ICU time 1.6-2.3 hr | 0.64 (0.40-1.01) |  | ED to ICU time 1.6-2.3 hr | 0.92 (0.76-1.11) |  |
| ED to ICU time 2.3-3.4 hr | 0.83 (0.61-1.14) |  | ED to ICU time 2.3-3.4 hr | 0.70 (0.58-0.86) *b* |  |
| ED to ICU time >3.4 hr | 0.81 (0.61-1.06) |  | ED to ICU time >3.4 hr | 0.64 (0.52-0.80) *b* |  |
|  |  |  |  |  |  |
| ‘’D1’’ ED to ICU time x Triage score 3 (yellow) |  |  | ‘’D2’’ ED to ICU time x Triage score 3 (yellow) |  |  |
| ED to ICU time <1.1 hr | Reference | p=0.29 | ED to ICU time <1.1 hr | Reference | p<0.01 |
| ED to ICU time 1.1-1.6 hr | 0.71 (0.22-2.33) |  | ED to ICU time 1.1-1.6 hr | 0.71 (0.41-1.22) |  |
| ED to ICU time 1.6-2.3 hr | 0.23 (0.06-0.82) *b* |  | ED to ICU time 1.6-2.3 hr | 0.78 (0.49-1.25) |  |
| ED to ICU time 2.3-3.4 hr | 0.49 (0.22-1.10) |  | ED to ICU time 2.3-3.4 hr | 0.45 (0.28-0.72) *b* |  |
| ED to ICU time >3.4 hr | 0.63 (0.33-1.19) |  | ED to ICU time >3.4 hr | 0.58 (0.37-0.89) *b* |  |
|  |  |  |  |  |  |
| ‘’E1’’ ED to ICU time x Triage score 2 (orange) |  |  | ‘’E2’’ ED to ICU time x Triage score 2 (orange) |  |  |
| ED to ICU time <1.1 hr | Reference | p=0.32 | ED to ICU time <1.1 hr | Reference | p=0.17 |
|  |  |  |  |  |  |
| eTable 9 odds ratios for hospital mortality in the academic hospital cohort and non-academic teaching hospital cohort adjusted for ED triage | | | | | |
|  |  |  |  |  |  |
| ED to ICU time 1.1-1.6 hr | 0.90 (0.62-1.30) |  | ED to ICU time 1.1-1.6 hr | 0.96 (0.75-1.23) |  |
| ED to ICU time 1.6-2.3 hr | 0.87 (0.62-1.22) |  | ED to ICU time 1.6-2.3 hr | 0.91 (0.71-1.16) |  |
| ED to ICU time 2.3-3.4 hr | 0.86 (0.61-1.19) |  | ED to ICU time 2.3-3.4 hr | 0.98 (0.78-1.24) |  |
| ED to ICU time >3.4 hr | 0.73 (0.53-1.01) |  | ED to ICU time >3.4 hr | 0.88 (0.69-1.13) |  |
|  |  |  |  |  |  |
| ‘’F1’’ ED to ICU time x Triage score 1 (red) |  |  | ‘’F2’’ ED to ICU time x Triage score 1 (red) |  |  |
| ED to ICU time <1.1 hr | Reference | p=0.05 | ED to ICU time <1.1 hr | Reference | p<0.01 |
| ED to ICU time 1.1-1.6 hr | 1.20 (0.98-1.48) |  | ED to ICU time 1.1-1.6 hr | 0.98 (0.82-1.17) |  |
| ED to ICU time 1.6-2.3 hr | 1.31 (1.07-1.62) *b* |  | ED to ICU time 1.6-2.3 hr | 1.07 (0.88-1.30) |  |
| ED to ICU time 2.3-3.4 hr | 1.37 (1.08-1.73) *b* |  | ED to ICU time 2.3-3.4 hr | 0.78 (0.61-1.01) |  |
| ED to ICU time >3.4 hr | 1.14 (0.85-1.54) |  | ED to ICU time >3.4 hr | 0.52 (0.34-0.79) *b* |  |
|  |  |  |  |  |  |
|  |  |  |  |  |  |
|  |  |  |  |  |  |
|  |  |  |  |  |  |
|  |  |  |  |  |  |

ED to ICU time = emergency department to intensive care unit time

*a* All models are adjusted for hospital

*b* p < 0.05.

Values represent the odds ratios and 95% CIs.

The p is analyzing whether ED to ICU time as a total factor is associated with the hospital, we used a Wald test for the ED to ICU variables

# eTable 10 odds ratios for ICU mortality in the overall cohort adjusted for APACHE-IV probability and ED triage

| **Model** | **ICU mortality *^a^*** | **p** | **Model** | **ICU mortality** | p-value |
| --- | --- | --- | --- | --- | --- |
| ‘’A’’ ED to ICU time |  |  |  |  |  |
| ED to ICU time <1.1 hr | Reference | p<0.01 |  |  |  |
| ED to ICU time 1.1-1.6 hr | 0.83 (0.76-0.92) *b* |  |  |  |  |
| ED to ICU time 1.6-2.3 hr | 0.75 (0.68-0.82) *b* |  |  |  |  |
| ED to ICU time 2.3-3.4 hr | 0.52 (0.47-0.58) *b* |  |  |  |  |
| ED to ICU time >3.4 hr | 0.33 (0.29-0.37) *b* |  |  |  |  |
|  |  |  |  |  |  |
| ‘’B1’’ ED to ICU time; APACHE-IV  probability |  |  | ‘’B2’’ ED to ICU time; and Triage score |  |  |
| ED to ICU time <1.1 hr | Reference | p=0.47 | ED to ICU time <1.1 hr | Reference | p<0.01 |
| ED to ICU time 1.1-1.6 hr | 0.94 (0.84-1.05) |  | ED to ICU time 1.1-1.6 hr | 0.97 (0.87-1.08) |  |
| ED to ICU time 1.6-2.3 hr | 0.94 (0.84-1.06) |  | ED to ICU time 1.6-2.3 hr | 0.97 (0.86-1.09) |  |
| ED to ICU time 2.3-3.4 hr | 0.94 (0.83-1.06) |  | ED to ICU time 2.3-3.4 hr | 0.81 (0.72-0.92) *b* |  |
| ED to ICU time >3.4 hr | 0.88 (0.77-1.01) |  | ED to ICU time >3.4 hr | 0.63 (0.55-0.72) *b* |  |
|  |  |  |  |  |  |
| ‘’C1’’ ED to ICU time x APACHE < 8.0% |  |  | ‘’C2’’ ED to ICU time x Triage score 4 (blue/green) |  |  |
| ED to ICU time <1.1 hr | Reference | p=0.41 | ED to ICU time <1.1 hr | Reference | p<0.01 |
| ED to ICU time 1.1-1.6 hr | 1.18 (0.54-2.56) |  | ED to ICU time 1.1-1.6 hr | 0.93 (0.76-1.15) |  |
| ED to ICU time 1.6-2.3 hr | 1.47 (0.72-2.98) |  | ED to ICU time 1.6-2.3 hr | 0.84 (0.69-1.02) |  |
| ED to ICU time 2.3-3.4 hr | 0.88 (0.41-1.89) |  | ED to ICU time 2.3-3.4 hr | 0.70 (0.58-0.85) |  |
| ED to ICU time >3.4 hr | 0.81 (0.38-1.73) |  | ED to ICU time >3.4 hr | 0.59 (0.49-0.61) |  |
|  |  |  |  |  |  |
| ‘’D1’’ ED to ICU time x APACHE 8.0%- 20.4  % |  |  | ‘’D2’’ ED to ICU time x Triage score 3 (yellow) |  |  |
| ED to ICU time <1.1 hr | Reference | p=0.31 | ED to ICU time <1.1 hr | Reference | p<0.01 |
| ED to ICU time 1.1-1.6 hr | 0.98 (0.80-1.21) |  | ED to ICU time 1.1-1.6 hr | 0.80 (0.46-1.39) |  |
| ED to ICU time 1.6-2.3 hr | 0.86 (0.70-1.07) |  | ED to ICU time 1.6-2.3 hr | 0.60 (0.36-0.99) *b* |  |
| ED to ICU time 2.3-3.4 hr | 0.91 (0.74-1.13) |  | ED to ICU time 2.3-3.4 hr | 0.37 (0.23-0.61) *b* |  |
| ED to ICU time >3.4 hr | 0.81 (0.65-1.01) |  | ED to ICU time >3.4 hr | 0.38 (0.25-0.59) *b* |  |
|  |  |  |  |  |  |
|  |  |  |  |  |  |

| eTable 10 odds ratios for ICU mortality in the overall cohort adjusted for APACHE-IV probability and ED triage | | | | | |
| --- | --- | --- | --- | --- | --- |
| ‘’E1’’ ED to ICU time x APACHE 20.4%- 55.4% |  |  | ‘’E2’’ ED to ICU time x Triage score 2 (orange) |  |  |
| ED to ICU time <1.1 hr | Reference | p=0.40 | ED to ICU time <1.1 hr | Reference | p<0.01 |
| ED to ICU time 1.1-1.6 hr | 0.92 (0.76-1.10) |  | ED to ICU time 1.1-1.6 hr | 0.95 (0.75-1.21) |  |
| ED to ICU time 1.6-2.3 hr | 0.94 (0.78-1.13) |  | ED to ICU time 1.6-2.3 hr | 0.89 (0.71-1.12) |  |
| ED to ICU time 2.3-3.4 hr | 0.96 (0.79-1.16) |  | ED to ICU time 2.3-3.4 hr | 0.89 (0.71-1.11) |  |
| ED to ICU time >3.4 hr | 1.09 (0.92-1.32) |  | ED to ICU time >3.4 hr | 0.69 (0.55-0.87) *b* |  |
|  |  |  |  |  |  |
| ‘’F1’’ ED to ICU time x APACHE >55.4 % |  |  | ‘’F2’’ ED to ICU time x Triage score 1 (red) |  |  |
| ED to ICU time <1.1 hr | Reference | p=0.42 | ED to ICU time <1.1 hr | Reference | p<0.01 |
| ED to ICU time 1.1-1.6 hr | 0.94 (0.80-1.09) |  | ED to ICU time 1.1-1.6 hr | 1.05 (0.92-1.21) |  |
| ED to ICU time 1.6-2.3 hr | 1.05 (0.89-1.22) |  | ED to ICU time 1.6-2.3 hr | 1.11 (0.96-1.29) |  |
| ED to ICU time 2.3-3.4 hr | 0.99 (0.83-1.19) |  | ED to ICU time 2.3-3.4 hr | 1.03 (0.87-1.23) |  |
| ED to ICU time >3.4 hr | 1.16 (0.93-1.46) |  | ED to ICU time >3.4 hr | 0.76 (0.59-0.98) *b* |  |

APACHE = Acute Physiology and Chronic Health Evaluation, ED to ICU time = emergency department to intensive care unit time

*a* All models are adjusted for hospital

*b* p < 0.05.

Values represent the odds ratios and 95% CIs.

The p is analyzing whether ED to ICU time as a total factor is associated with the ICU mortality, we used a Wald test for the ED to ICU variables

# eTable 11 odds ratios for hospital mortality in the overall, academic hospital cohort and non-academic teaching hospital cohort of patients treated for an cardiac arrest adjusted for APACHE III

| **Model**  **– Cardiac arrest all patients (n=3,818) *^a^*** | **Hospital mortality** | **p** | **Model *^a^*** | **Hospital mortality** | p-value |
| --- | --- | --- | --- | --- | --- |
| ‘’A’’ ED to ICU time; |  |  |  |  |  |
| ED to ICU time <1.1 hr | Reference | p=0.17 |  |  |  |
| ED to ICU time 1.1-1.6 hr | 0.95 (0.80 – 1.13) |  |  |  |  |
| ED to ICU time 1.6-2.3 hr | 0.88 (0.74 – 1.05) |  |  |  |  |
| ED to ICU time 2.3-3.4 hr | 0.90 (0.73 – 1.11) |  |  |  |  |
| ED to ICU time >3.4 hr | 1.32 (0.93 – 1.87) |  |  |  |  |
|  |  |  |  |  |  |
| ‘’B1’’ ED to ICU time; APACHE III score |  |  | ‘’B2’’ ED to ICU time; Triage score |  |  |
| ED to ICU time <1.1 hr | Reference | p=0.03 | ED to ICU time <1.1 hr | Reference | p=0.16 |
| ED to ICU time 1.1-1.6 hr | 0.86 (0.71 - 1.04) |  | ED to ICU time 1.1-1.6 hr | 0.95 (0.80 - 1.12) |  |
| ED to ICU time 1.6-2.3 hr | 0.83 (0.69 - 1.00) |  | ED to ICU time 1.6-2.3 hr | 0.87 (0.73 - 1.04) |  |
| ED to ICU time 2.3-3.4 hr | 0.86 (0.68 - 1.08) |  | ED to ICU time 2.3-3.4 hr | 0.89 (0.73 – 1.10) |  |
| ED to ICU time >3.4 hr | 1.47 (0.99 – 2.18) |  | ED to ICU time >3.4 hr | 1.30 (0.92 – 1.86) |  |
|  |  |  |  |  |  |
| **Model – Cardiac arrest academic patients (n=1,872) *^a^*** | **Hospital mortality** | **p** | **Model *^a^*** | **Hospital mortality** | p |
| ‘’A’’ ED to ICU time; |  |  |  |  |  |
| ED to ICU time <1.1 hr | Reference | P=0.006 |  |  |  |
| ED to ICU time 1.1-1.6 hr | 1.37 (1.03 – 1.81) *b* |  |  |  |  |
| ED to ICU time 1.6-2.3 hr | 1.47 (1.12 – 1.93) *b* |  |  |  |  |
| ED to ICU time 2.3-3.4 hr | 1.57 (1.16 – 2.12) *b* |  |  |  |  |
| ED to ICU time >3.4 hr | 2.01 (1.31 – 3.09) *b* |  |  |  |  |
|  |  |  |  |  |  |
| ‘’B1’’ ED to ICU time; APACHE III score |  |  | ‘’B2’’ ED to ICU time; Triage score |  |  |
| ED to ICU time <1.1 hr | Reference | P<0.001 | ED to ICU time <1.1 hr | Reference | p=0.004 |
| ED to ICU time 1.1-1.6 hr | 1.48 (1.08 – 2.02) *b* |  | ED to ICU time 1.1-1.6 hr | 1.39 (1.05 – 1.84) *b* |  |
| ED to ICU time 1.6-2.3 hr | 1.69 (1.25 – 2.29) *b* |  | ED to ICU time 1.6-2.3 hr | 1.48 (1.12 – 1.95) *b* |  |
| ED to ICU time 2.3-3.4 hr | 1.87 (1.33 - 2.62) *b* |  | ED to ICU time 2.3-3.4 hr | 1.58 (1.17 – 2.15) *b* |  |
| ED to ICU time >3.4 hr | 2.94 (1.80 – 4.78) *b* |  | ED to ICU time >3.4 hr | 2.09 (1.35 – 3.22) *b* |  |

| eTable 11 odds ratios for hospital mortality in the overall, academic hospital cohort and non-academic teaching hospital cohort of patients treated for an cardiac arrest adjusted for APACHE III | | | | | |
| --- | --- | --- | --- | --- | --- |
| **Model – Cardiac arrest non-academic patients (n=1,946)** | **Hospital mortality** | **p** | **Model *^a^*** | Hospital mortality | p |
| ‘’A’’ ED to ICU time; |  |  |  |  |  |
| ED to ICU time <1.1 hr | Reference | p<0.001 |  |  |  |
| ED to ICU time 1.1-1.6 hr | 0.81 (0.65 – 1.02) |  |  |  |  |
| ED to ICU time 1.6-2.3 hr | 0.63 (0.50 – 0.80) *b* |  |  |  |  |
| ED to ICU time 2.3-3.4 hr | 0.58 (0.42 – 0.80) *b* |  |  |  |  |
| ED to ICU time >3.4 hr | 0.95 (0.48 – 1.91) |  |  |  |  |
|  |  |  |  |  |  |
| ‘’B1’’ ED to ICU time; and **APACHE III**  score |  |  | ‘’B2’’ ED to ICU time; and Triage score |  |  |
| ED to ICU time <1.1 hr | Reference | p<0.001 | ED to ICU time <1.1 hr | Reference | p<0.001 |
| ED to ICU time 1.1-1.6 hr | 0.68 (0.54 – 0.88) *b* |  | ED to ICU time 1.1-1.6 hr | 0.81 (0.65 – 1.01) |  |
| ED to ICU time 1.6-2.3 hr | 0.55 (0.42 – 0.71) *b* |  | ED to ICU time 1.6-2.3 hr | 0.62 (0.49 – 0.79) *b* |  |
| ED to ICU time 2.3-3.4 hr | 0.49 (0.34 - 0.70) *b* |  | ED to ICU time 2.3-3.4 hr | 0.55 (0.40 – 0.77) *b* |  |
| ED to ICU time >3.4 hr | 0.74 (0.33 – 1.64) |  | ED to ICU time >3.4 hr | 0.89 (0.44 – 1.80) |  |

APACHE = Acute Physiology and Chronic Health Evaluation, ED to ICU time = emergency department to intensive care unit time

*a* All models are adjusted for hospital

*b* p < 0.05.

Values represent the odds ratios and 95% CIs.

The p is analyzing whether ED to ICU time as a total factor is associated with the hospital mortality, we used a Wald test for the ED to ICU variables

#
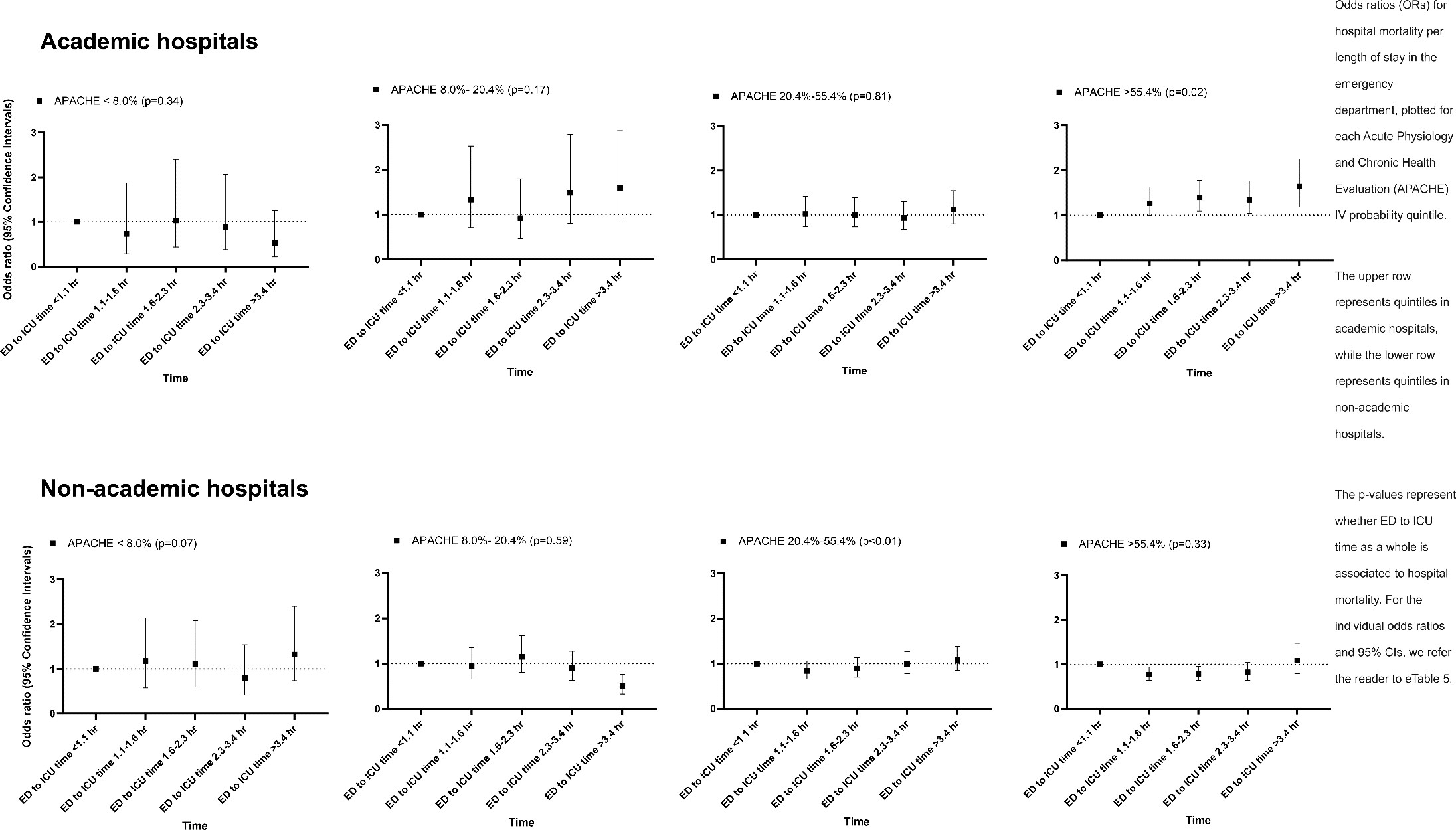
eFigure 1 odds ratios for hospital mortality per length of stay in the emergency department, for each Acute Physiology and Chronic Health Evaluation quintile

#
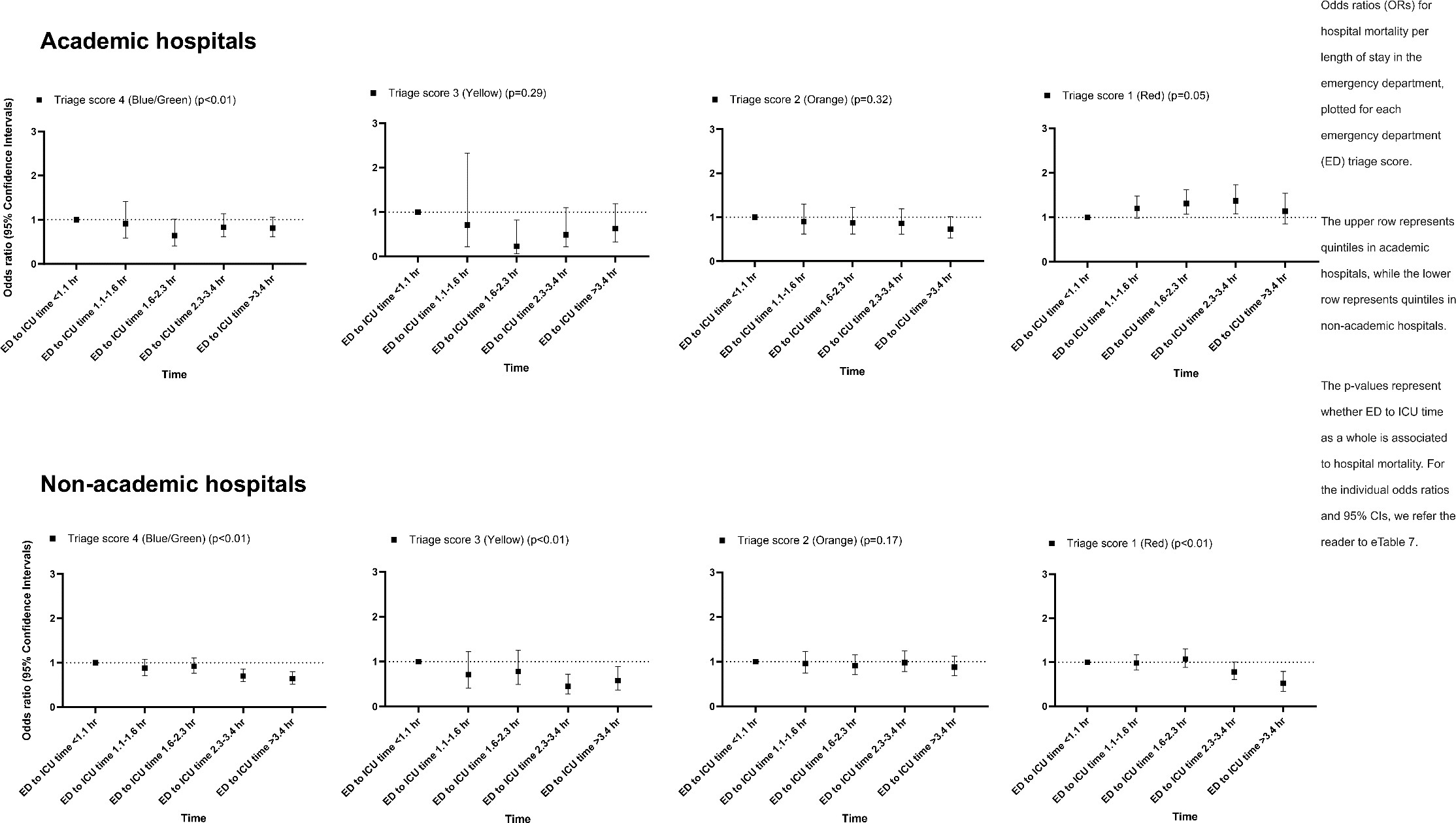
eFigure 2 odds ratios for hospital mortality per length of stay in the emergency department, for each ED triage score
